# Capture and light-induced release of antibiotics by an azo dye polymer

**DOI:** 10.1038/s41598-020-60245-6

**Published:** 2020-02-24

**Authors:** Stephen Atkins, Alysa Chueh, Taylor Barwell, Jean-Michel Nunzi, Laurent Seroude

**Affiliations:** 1 Department of Biology, Queen’s University, Kingston, ON K7L-3N6 Canada; 2 Department of Chemistry, Queen’s University, Kingston, ON K7L-3N6 Canada; 3 Department of Physics, Engineering Physics and Astronomy, Queen’s University, Kingston, ON K7L-3N6 Canada

**Keywords:** Photobiology, Polymers, Drug delivery, Nanomedicine

## Abstract

The isomerisation of azo dyes can induce conformational changes which have potential applications in medicine and environmental protection. We developed an agar diffusion assay to test the capture and release of biologically active molecules from an azo electro-optic polymer, Poly (Disperse Red 1 methacrylate) (DR1/PMMA). The assay monitors the growth of bacteria placed in soft agar under a glass coverslip. Antibiotics can then be applied on the coverslip resulting in the clearance of the area under the coverslip due to growth inhibition. This assay demonstrates that DR1/PMMA is able to capture either tetracycline or ampicillin and the relative amount of DR1/PMMA required for capture was determined. Finally, the active antibiotics can be released from DR1/PMMA by exposure to green laser light. Exposure to white light from a torch or to heat does not release the antibiotic.

## Introduction

Materials containing azo-benzene chromophores exhibit a range of photo-responsive properties^1^. Under various light irradiation, -N = N- azo groups change from their *trans* to their *cis* conformations. The area of motions generated by azobenzene isomerization in polymer materials range from slight reorientations of the chromophore to mass motion of the polymer as well as, surface relief grating, and a variety of nonlinear optical effects^2^. Poly (Disperse Red 1 methacrylate) (DR1/PMMA) is a polymer with a -N = N- azo group attached to the backbone MMA monomers through covalent bonds. DR1/PMMA can occur in two different geometries, a linear stretched *trans* configuration and an angular *cis* conformation. The polymer exhibits good thermal and temporal stabilities with a high glass transition temperature^3^, *T*_*g*_ = 91 °C^4^.

In this report, a biological assay is designed to test the ability of DR1/PMMA to capture and release biologically active molecules. Two broadly used antibiotics, ampicillin and tetracycline are tested. Tetracycline is the simplest member of the “tetracycline” class of antibiotics defined by the presence of the DCBA naphthacene core containing four aromatic rings^5^. Tetracycline binds to the 30 S ribosomal subunit and leads to the inhibition of protein translation by preventing tRNAs docking. Ampicillin belongs to the beta-lactam class of antibiotics defined by the presence of a highly reactive 3-carbon and 1-nitrogen ring^6^. Ampicillin interferes with the synthesis of peptidoglycan necessary for the bacterial cell wall. Ampicillin and tetracycline are polar molecules that can form hydrogen bonds with DR1/PPMA^7,8^.

## Results

### Agar diffusion assay

An agar diffusion assay was first developed to be able to monitor the growth of bacteria placed in soft agar under a glass coverslip. Four Escherichia coli strains commonly used in molecular biology were used (Fig. S1). The presence of tetracycline and ampicillin will be respectively detected with the tetracycline-sensitive (tet^s^) TB1 and HB101 strains and ampicillin-sensitive (amp^s^) DH5α and XL1Blue strains. TB1 and HB101 have been transformed with the pUAST and pBSGFP plasmids conferring resistance to ampicillin. XL1Blue and DH5α respectively contains a F’ factor and the pHC60 plasmid conferring resistance to tetracycline. The resistance to tetracycline and ampicillin eliminates the occurrence of antibiotic-sensitive contaminants and facilitates the assay for experimentalists unacquainted with aseptic techniques. Additionally the tet^s^ amp^r^ HB101:pBSGFP and the amp^s^ tet^r^ DH5α:pHC60 strains produce GFP for alternative visualization by fluorescence. Because the assay will be standardized by inoculating the soft agar medium with a fixed amount of a liquid culture, the growth of each strain in a liquid culture was examined by measuring the optical density at 600 nm. Since the optical density is affected by cell size and cannot differentiate between dead and alive cells, the number of cells alive was measured simultaneously by serial dilution plating.

**Figure 1:**
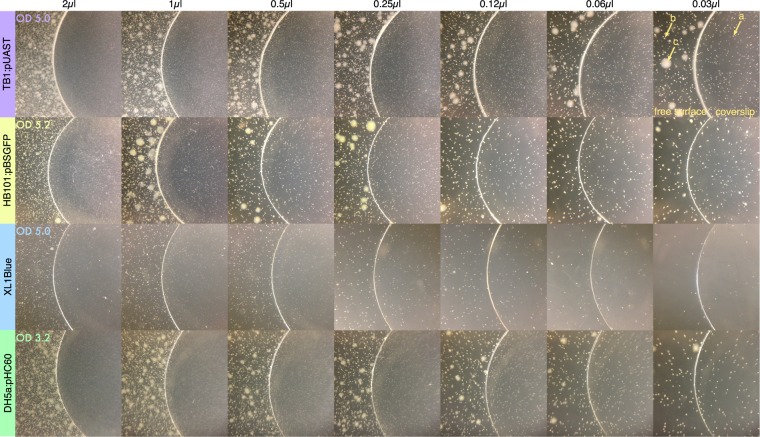
Influence of cell density on growth under coverslips. The optical density at 600 nm of the liquid cultures is indicated for each bacterial strain (first picture of each row). The soft-agar was inoculated with aliquots of 1/100 dilutions of the cultures and the equivalent amount of undiluted cultures is indicated at the top of each column. The top-right image reveals the layout of the assay: free surface and coverslip. Arrows indicate (**a**) a colony grown under the coverslip; (**b**) a colony grown under the free surface, deep into the agar; (**c**) a colony grown under the free surface, on top of the agar where oxygen is more abundant.

Next soft agar plates were inoculated with different amounts of liquid cultures of each strain and the bacterial growth occurring under a clean glass coverslip was qualitatively assessed for the easiest visualization (Fig. 1). The plates with higher cell density clearly show that the biggest colonies grew on the surface of the top agar while the colonies below the surface were smaller and the smallest colonies were always found under the coverslip as expected from the decreased oxygen availability. As the cell density decreases, the colonies under the coverslip have obviously increased their size and are now similar to the colonies outside of the coverslip thereby greatly improving visualization with the naked eye.

**Figure 2:**
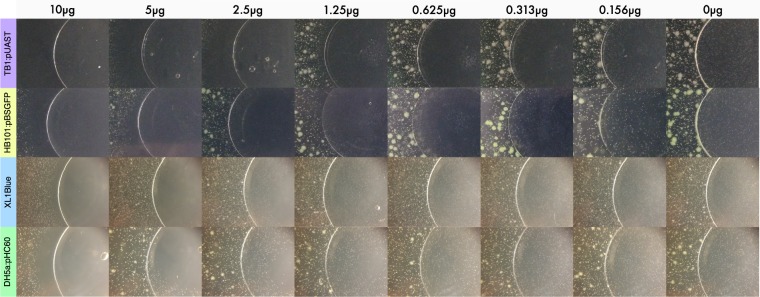
Determination of the amount of antibiotic in the agar diffusion assay. The mass of antibiotic applied to each coverslip is indicated at the top of each column. The top two rows (TB1 and HB101 strains) used tetracycline coverslips while the bottom two rows (XL1Blue and DH5α) used ampicillin coverslips. See Fig. 1 to identify colonies.

Next the amount of antibiotic required to inhibit bacterial growth was determined. 10 µl aliquots of ampicillin or tetracycline dissolved in dimethylformamide (DMF) at appropriate concentrations were pipetted on clean glass coverslips and the solvent was evaporated. This process generated series of coverslips with different amounts of antibiotics that were then tested on the bacterial strains (Fig. 2). The tet^s^ strains show that 0.313 µg of tetracycline is the lower amount required to observe some bacterial clearance but 1.25 µg is needed for complete clearance and higher amounts extend the clearance zone well outside the coverslip area. It is noticeable that HB101 is slightly less sensitive than TB1. The amp^s^ strains show that 1.25 µg of ampicillin is required to observe some clearance but 5 µg is needed for complete clearance. These results agree with the 12.5–25 µg/ml tetracycline and 50–100 µg/ml ampicillin concentrations routinely used to select antibiotic-resistant *E*. *coli*.

### DR1/PMMA can capture antibiotics and restore bacterial growth

**Figure 3:**
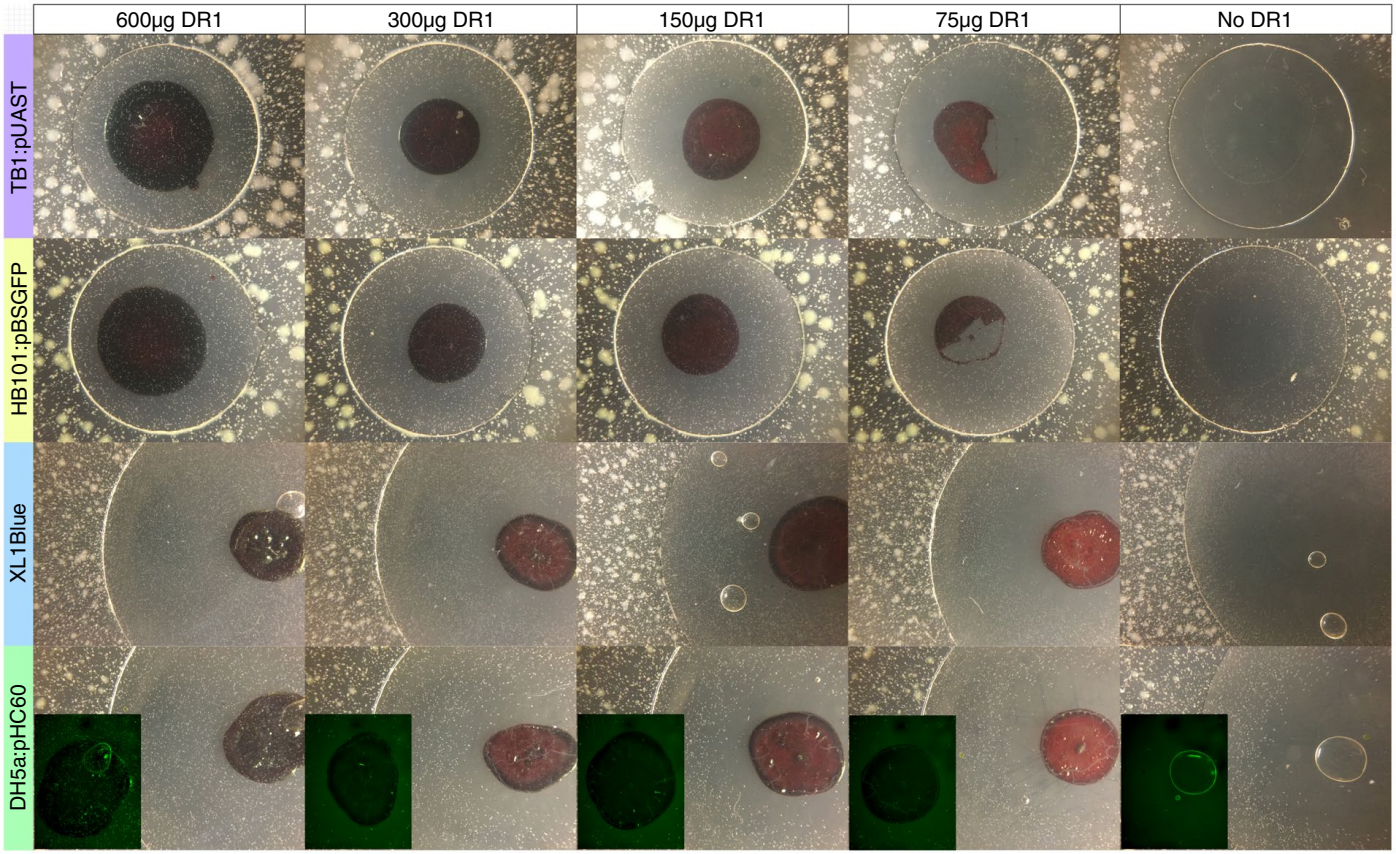
Antibiotic capture with DR1/PMMA. All coverslips have been prepared with 0.625 µg tetracycline (top two rows) or 2.5 µg ampicillin (bottom two rows) supplemented with the mass of DR1/PMMA indicated at the top of each column. Inset pictures in the bottom row show alternative visualization using GFP fluorescence. See Fig. 1 to identify colonies.

Although the chemical structures of ampicillin and tetracycline allow hydrogen binding with DR1/PMMA, it is not possible to mathematically determine the amount of DR1/PMMA needed since the number of antibiotic molecules that can bind per structural unit is unknown. Therefore, the ability to capture these antibiotics and the appropriate molecular ratio was determined experimentally with the agar diffusion assay. After verification that it does not affect bacterial growth (Fig. S2), different amounts of DR1/PMMA were added to 0.625 µg tetracycline or 2.5 µg ampicillin and the resulting coverslips were tested (Fig. 3). Compared to the control coverslips, the addition of DR1/PMMA reduced the clearance zone caused by either antibiotic. However, both antibiotics required 600 µg DR1/PMMA to fully restore bacterial growth. These observations show that DR1/PMMA is able to capture both antibiotics and capture is more efficient with ampicillin than tetracycline.

### Antibiotics can be released with green light

**Figure 4:**
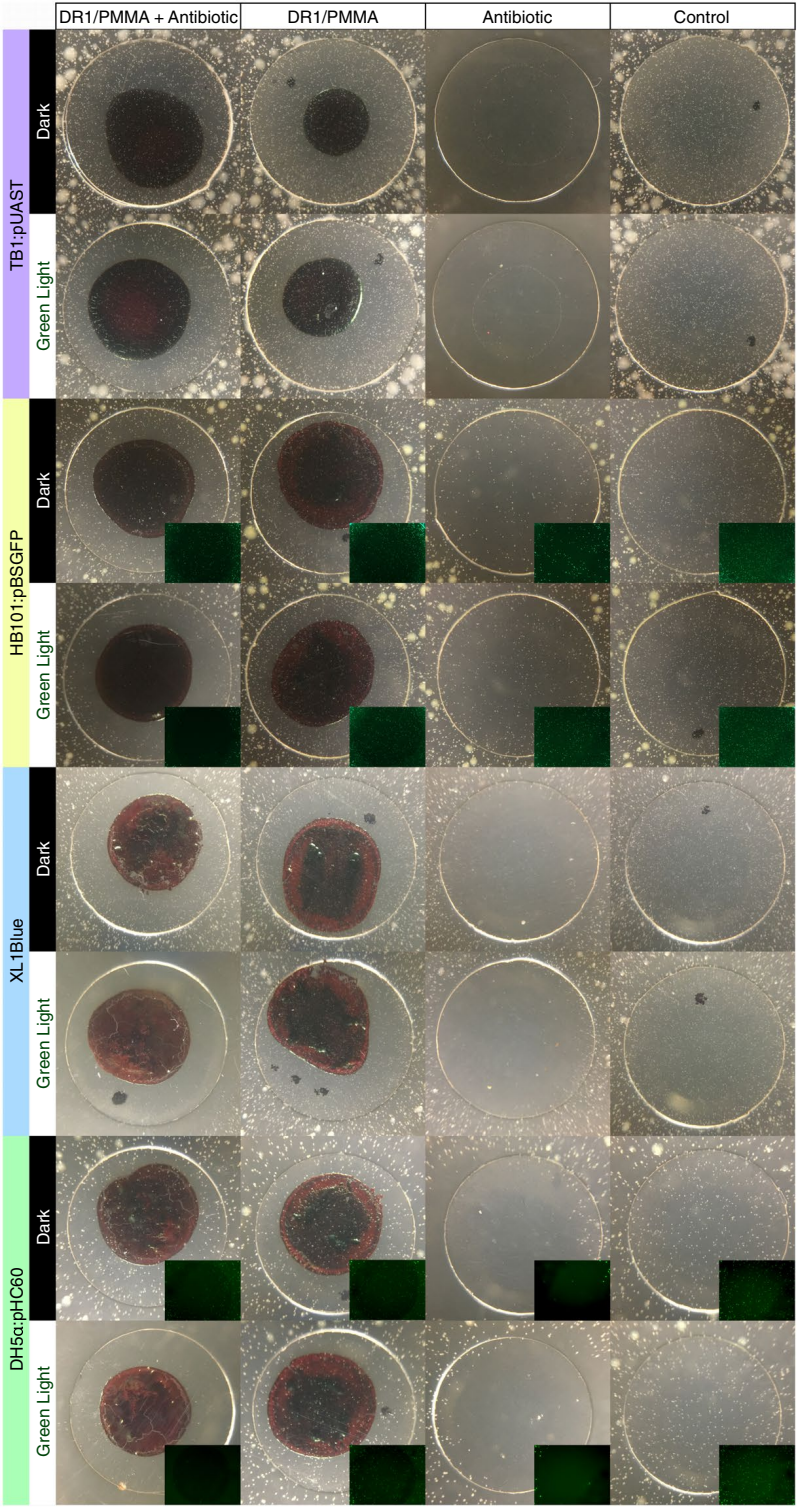
Antibiotic release by exposure to green light. Negative control coverslips (Control) have neither antibiotic nor DR1/PMMA. 0.625 µg tetracycline (top four rows) or 2.5 µg ampicillin (bottom four rows) was applied to the positive control coverslips (Antibiotic). 600 µg DR1/PMMA was applied to no antibiotic control coverslips (DR1/PMMA). 0.625 µg tetracycline (top four rows) or 2.5 µg ampicillin (bottom four rows), and 600 µg DR1/PMMA was applied to the experimental coverslips (DR1/PMMA + Antibiotic). See Fig. 1 to identify colonies.

Once it has been established that DR1/PMMA protects E. coli from either antibiotic, the agar diffusion assay can be used to test whether the antibiotic can be released and has retained its biological activity. DR1 isomerization is typically achieved by exposure to wavelengths between 488 nm and 532 nm^1,9^. It has been known for a long time that visible light above 400 nm can affect respiration, ATPase activity and decreases ATP level of the ML 308 *E*. *coli* strain^10^. More recently it has been observed that exposure to green light can affect the viability and growth of the MG 1655 *E*. *coli* strain^11^. Therefore, the effect of green light on the agar diffusion assay was tested and found not to affect the growth of the four *E*. *coli* strains (Fig. S3). Once exposed to green light, the DR1/PMMA + antibiotic coverslips restore the clearance zone demonstrating that both antibiotics can be released and are still active (Fig. 4). The exposed DR1/PMMA + ampicillin coverslips are almost identical to the positive controls indicating complete release whereas the DR1/PMMA+ tetracycline coverslips suggest that the release is incomplete or/and that tetracycline lost some activity. Importantly release is not observed when the coverslips are exposed to heat (Fig. S4) or to white light (Fig. S5). Although white light includes green wavelengths, the intensity is insufficient as preliminary observations indicated that the release of ampicillin is not detectable after one-hour exposure and does require overnight exposure (Fig. S6).

## Discussion

The light-induced release of antibiotics by the DR1/PMMA polymer can be attributed to a manifold of molecular processes. The bacteria in our experiment is not expected to be the drug carrier^12^, but water could dissolve into the water insoluble-polymer under illumination and wash the antibiotic out of the polymer, like DR1-molecules dissolved into a cellulosic polymer host under light^13^. However, the experiment did not show any swelling of the neat polymer under illumination. The photoinduced movement of the DR1-chromophores could also be suspected to expel the antibiotic from the host. Molecular dynamics simulations indeed suggest that the repeated isomerization of DR1 molecules induces an accelerated diffusion of the host^14^. However, an experiment performed using a molecular DR1-glass showed that phase separation does not happen under illumination^15^. The spinodal decomposition of linear polymers is a well-documented entropy driven event^16^. We suggest that a photo-induced spinodal decomposition happens during illumination^17^, which expels the antibiotic from the entangled DR1/PMMA polymer chains into the water of the assay.

## Conclusion

The ability to control the activity of biological molecules as well as the ability to relieve the control is critical for a multitude of applications in medical and environmental sciences. It is widely recognized that antibiotics are less effective mainly because of the evolution of resistant bacteria. The possibility of releasing an antibiotic only where needed allows increasing therapeutic doses without exposing the whole microbiota as well as minimizing risks of side effects. The trapping of antibiotics has the additional advantage to minimize the impact of excreted molecules and discarded pills.

The agar diffusion assay is a very simple and cheap method to quickly assess the ability to capture and release antibiotics. In this report, the assay was used to demonstrate that ampicillin and tetracycline can be captured by DR1/PMMA and can subsequently be released by exposure to green light. It appeared that the capture and release with this polymer is more efficient for ampicillin than tetracycline but the ease of the assay allows screening through any polymers or molecules.

## Methods

### Bacteria strains

XL1Blue (recA1 endA1 gyrA96 thi-1 hsdR17 supE44 relA1 lac [F’ proAB lacI^q^ ∆(lacZ)M15 Tn10 (Tet^r^)]) has been obtained from Stratagene. TB1:pUAST (ara ∆(lac proAB) [Φ80dlac ∆(lacZ)M15] rpsL (Str^r^) thi hsdR: pUAST (Amp^r^)) and DH5α:pHC60 (recA1 endA1 gyrA96 thi-1 hsdR17 relA1 [Φ80dlac ∆(lacZ)M15] ∆(lacZYA argF)U169:pHC60 (Tet^r^)) have been previously described^18,19^. HB101:pBSGFP: supE44 ara14 galK2 lacY1 ∆(gpt-proA)62 rpsL20 (Str^r^) xyl-5 mtl-1 recA13 ∆(mcrC^−^ mr^r^) hsdS-(r^−^ m^−^):pBSGFP (Amp^r^) was obtained by transformation of HB101 with the pBSGFP plasmid (a pBluescript plasmid expressing GFP). All strains were cultured on 2TY (1% yeast extract, 1.6% tryptone, 0.5% NaCl) agar (1.5%) plates or broth supplemented with 25 µg/ml tetracycline (tet^r^ strains) or 100 µg/ml ampicillin (amp^r^ strains).

### Solutions and coverslips

Poly (Disperse Red 1 methacrylate) (DR1/PMMA) (Sigma cat#579009), ampicillin (BioShop cat#AMP201) and tetracycline (BioShop cat#TET701) solutions and dilutions were prepared using dimethylformamide (DMF) (Fisher) as the solvent. Dilutions were prepared by 2-fold serial dilutions of 100 mg/ml ampicillin, 25 mg/ml tetracycline and 60 mg/ml DR1/PMMA stock solutions. 10 µl of the appropriate solutions were pipetted onto circle glass coverslips (Fisher cat#12-545-101 or Ultident cat#170-C12MM) and air dried at room temperature in the dark for a minimum of 16 h. Mixture solutions were obtained by mixing antibiotic and DR1/PMMA solutions or by dissolving the desired amount of DR1/PMMA in the antibiotic solution.

### Agar diffusion assay

2TY soft agar (2TY supplemented with 7 g/l agar) was autoclaved and cooled down to 47 °C before adding ampicillin (100 µg/ml final, amp^r^ strains) or tetracycline (25 µg/ml final, tet^r^ strains), and the desired number of bacteria from a fresh or overnight culture. The optical density of cultures was measured at 600 nm (always between 2 and 6) and a 100-fold dilution of the cultures were prepared. Based on the results presented in Fig. 1, 6 µl (TB1, HB101 and DH5α strains) or 25 µl (XL1Blue strain) of the culture dilution was added per 15 ml of soft agar. 15 ml of the soft agar/antibiotic/bacteria solution was then poured in 10 cm plastic petri dishes. Once the soft agar has jellified, coverslips were placed atop the surface. It is important not to move the coverslips once placed as it was observed that the antibiotic from control coverslips diffuse in the soft agar almost instantaneously (after a slight move of the coverslip, the growth inhibition zone remained centered to the original position as can be seen for the DH5α strain antibiotic controls in Fig. 4). Plates were incubated overnight (12 to 18 h) in a 37 °C bacterial incubator. Dark plates were placed inside a black box while exposed plates had a green laser light (532 nm 50 mW green laser diode module, eBay) positioned overtop the coverslips.

## Supplementary information

Supplementary information.
