# Supplementary material for: Capture and light-induced release of antibiotics by an azo dye polymer
Source: Sci Rep. 2020 Feb 24;10:3267. doi: 10.1038/s41598-020-60245-6 (PMC7039927; doi:10.1038/s41598-020-60245-6)
Supplement: Supplementary file 1 — Supplementary information. [file 41598_2020_60245_MOESM1_ESM.pdf]

Supplemental Data

Capture and light-induced release of antibiotics by an azo dye.

Stephen Atkins[1], Alysa Chueh[2], Taylor Barwell[1], Jean-Michel Nunzi[2] and Laurent Seroude[1]

Department of Chemistry[2], Department of Biology[1], Queen's University, Kingston, ON, Canada

Content

Figure S1: Escherichia coli strains used in this study. 2
Figure S2: DR1/PMMA does not affect growth. 3
Figure S3: Green light does not affect growth 4
Figure S4: Antibiotics are not released by heat. 5
Figure S5: Tetracyline cannot be released with white light. 6
Figure S6: Timing of release of ampicillin 7

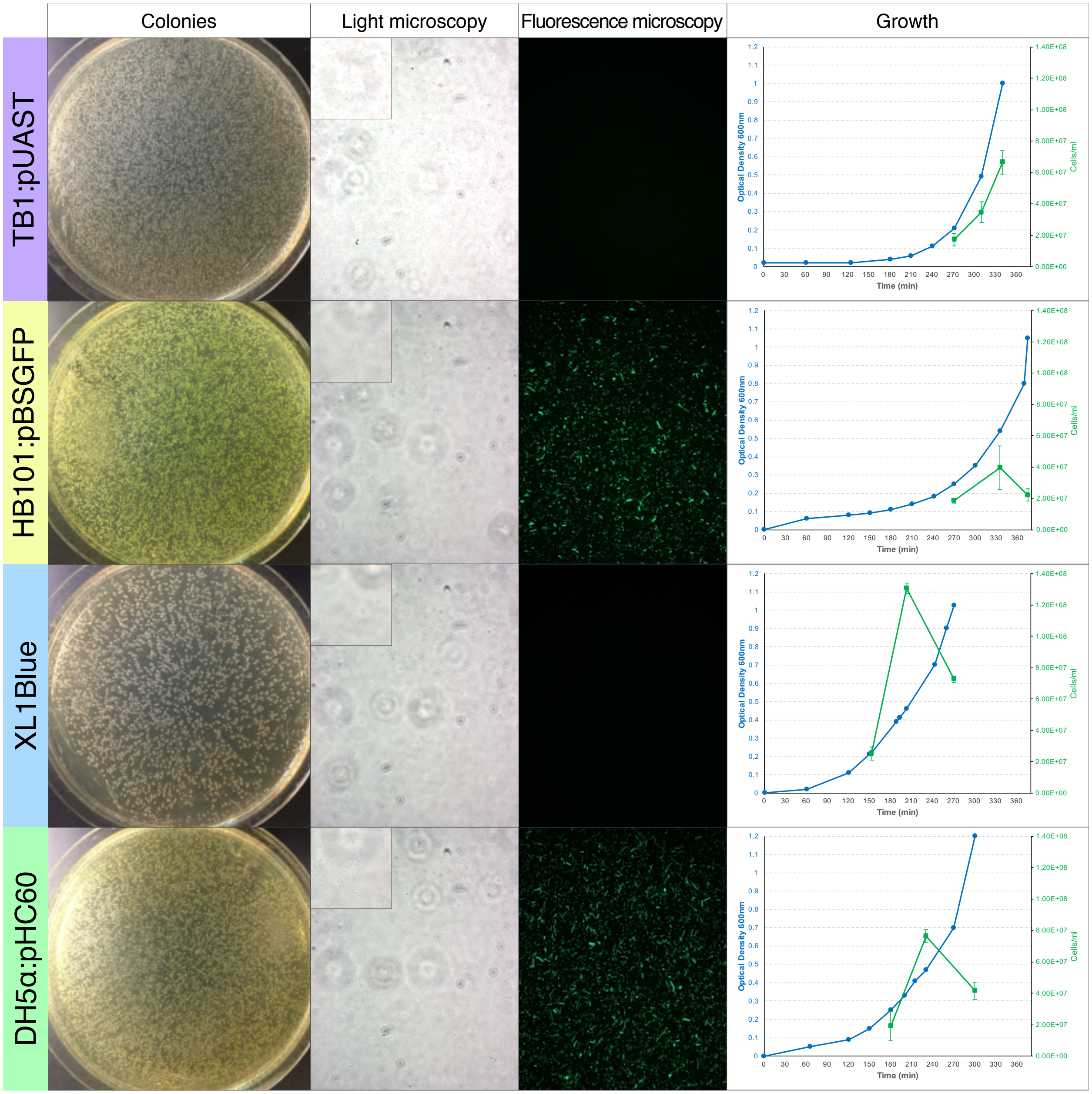

Figure S1: *Escherichia coli* strains used in this study.

The first column shows the appearance of colonies from each strain growing on 2TY agar. The GFP producing strains HB101:pBSGFP and DH5α:pHC60 exhibit colonies with a different color (green or slightly green). The second column shows the appearance of the cells with a light microscope (400x magnification, inset: digital zoom of subarea). The third column shows the appearance of the same cells with fluorescence microscopy. The fourth column shows the growth kinetics as the change in optical density and cell numbers as a function of time. Strains were cultured in 36ml 2TY supplemented with ampicillin or tetracycline in 250ml flasks under constant shaking in a 37˚C water-bath. Cultures were inoculated by 1/100 dilution of an overnight culture. Samples were periodically removed and the optical density at 600nm measured. When OD at 600nm reached approximately 0.2 (early exponential phase), 0.5 (mid-exponential phase) and 1.0 (late exponential phase), three to four samples of the cultures were plated at multiple serial dilutions and the number of colonies obtained after overnight incubation of the plates at 37˚C was used to determine the number of viable cells in the culture (error bars: ±SD).

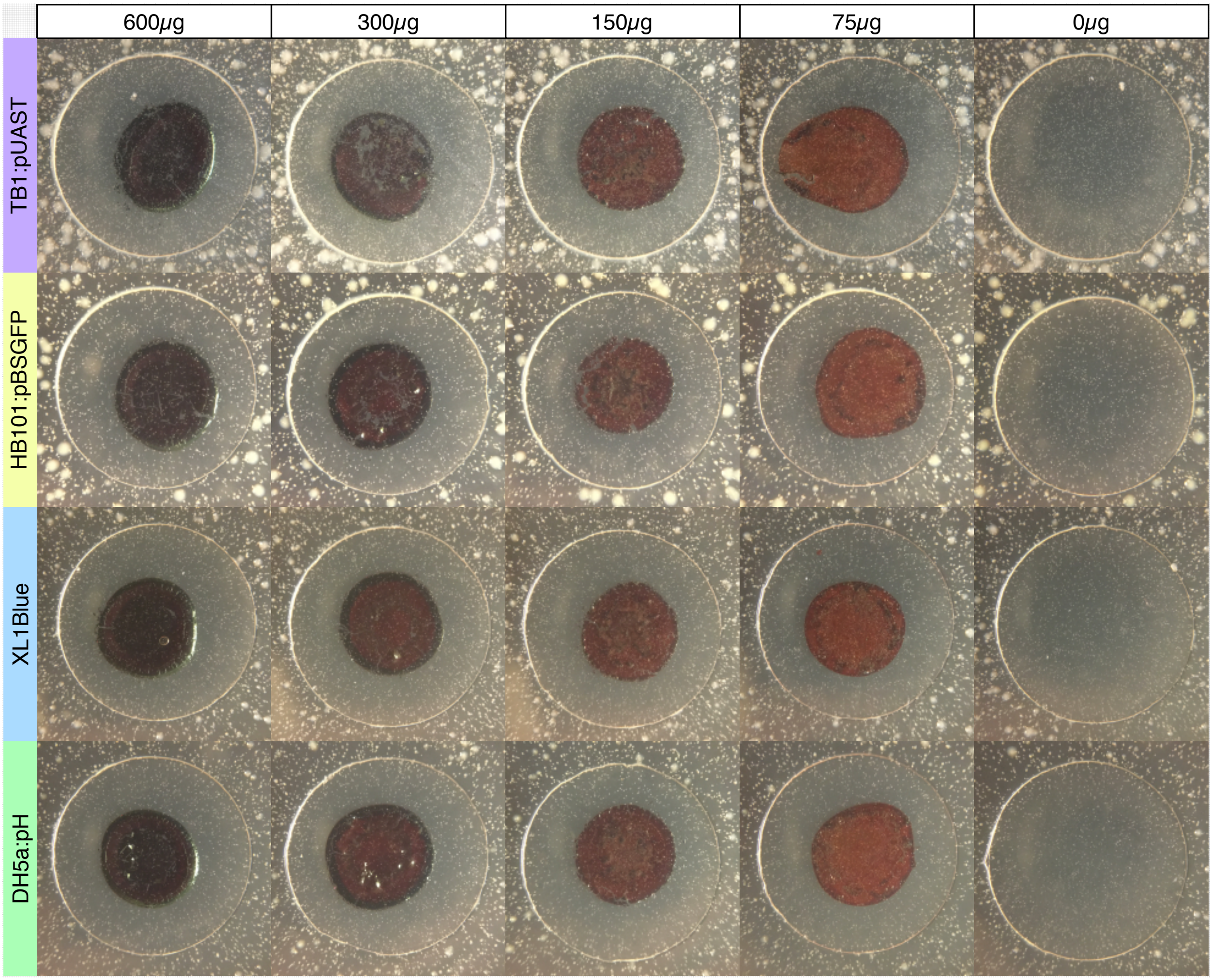

Figure S2: DR1/PMMA does not affect growth.

The agar diffusion assay was performed with coverslips with different amounts of DR1/PMMA (indicated at the top of each column). Plates were incubated overnight at 37˚C in the dark. Each strain was tested independently. No strain indicated sensitivity to DR1 at any concentration.

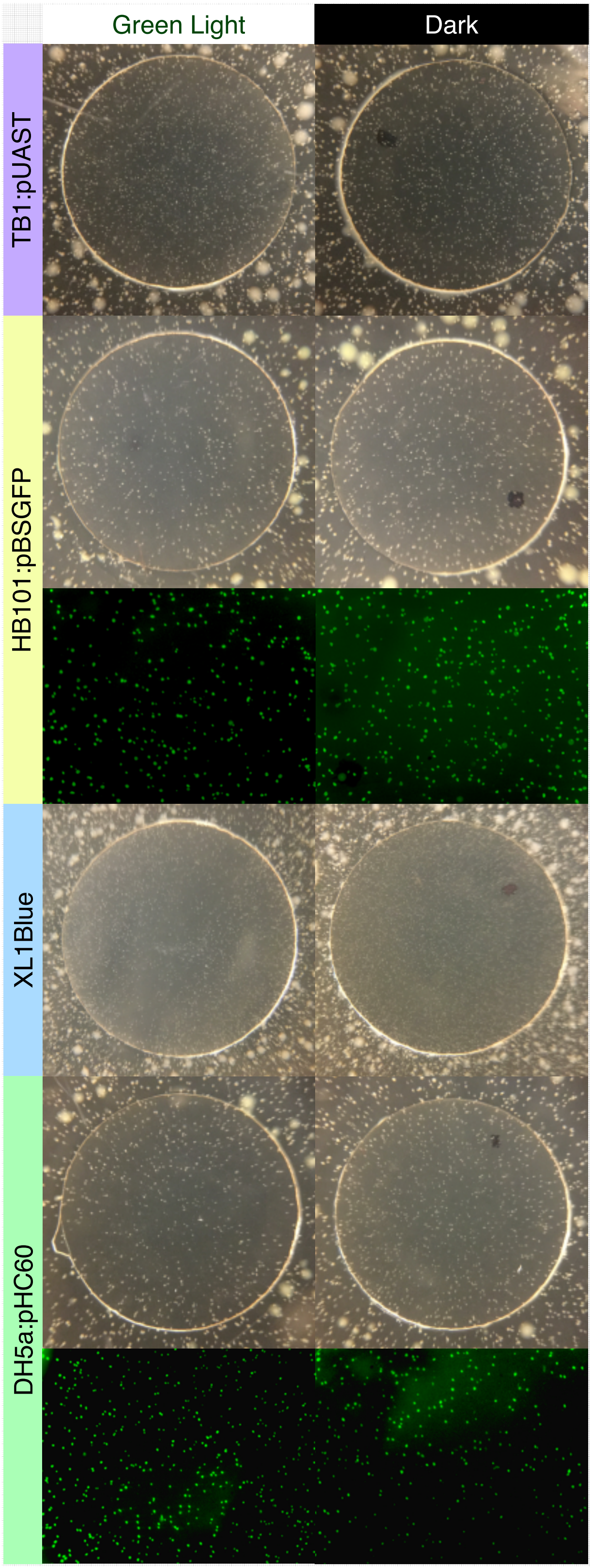

Figure S3: Green light does not affect growth

The agar diffusion assay was performed with blank coverslips Effect of green light on colony growth. Plates were incubated overnight in the dark (Dark) or with the green light directly overtop of the coverslip (Green light). The center of the coverslips was also visualized with GFP fluorescence for the GFP producing strains. The green light exposure show none (TB1, DH5α) or negligible effects (XL1Blue, HB101).

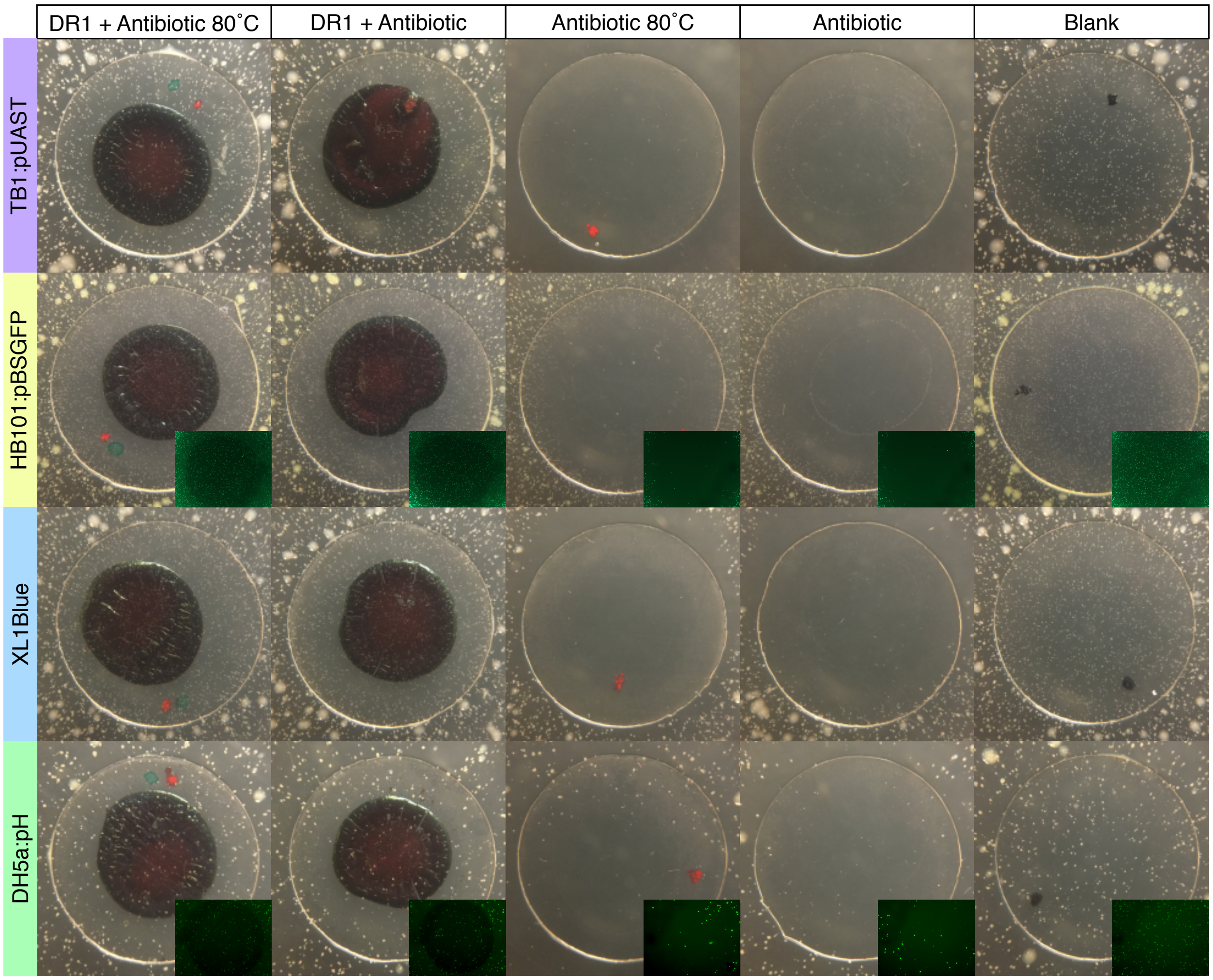

Figure S4: Antibiotics are not released by heat.

The agar diffusion assay was performed in the dark with DR1/PMMA + ampicillin (bottom two rows) or + tetracycline (top two rows) coverslips (DR1 + Antibiotic) and positive control coverslips with only ampicillin or tetracycline (Antibiotic). Coverslips were heated at 80˚C for 10 minutes in a vacuum oven and immediately placed atop the agar (80˚C). Antibiotic coverslips were heated and it was observed that ampicillin and tetracycline remained effective after heating. They were plated after cooling for 5 minutes at room temperature. The DR1 plus antibiotic coverslips were applied immediately out of the oven without a cooling period. There is no observable difference between the heated and non-heated coverslips and they are undistinguishable from the negative control coverslip (Blank). The center of the coverslips was also visualized with GFP fluorescence for the GFP producing strains (insets row 2 and 4).

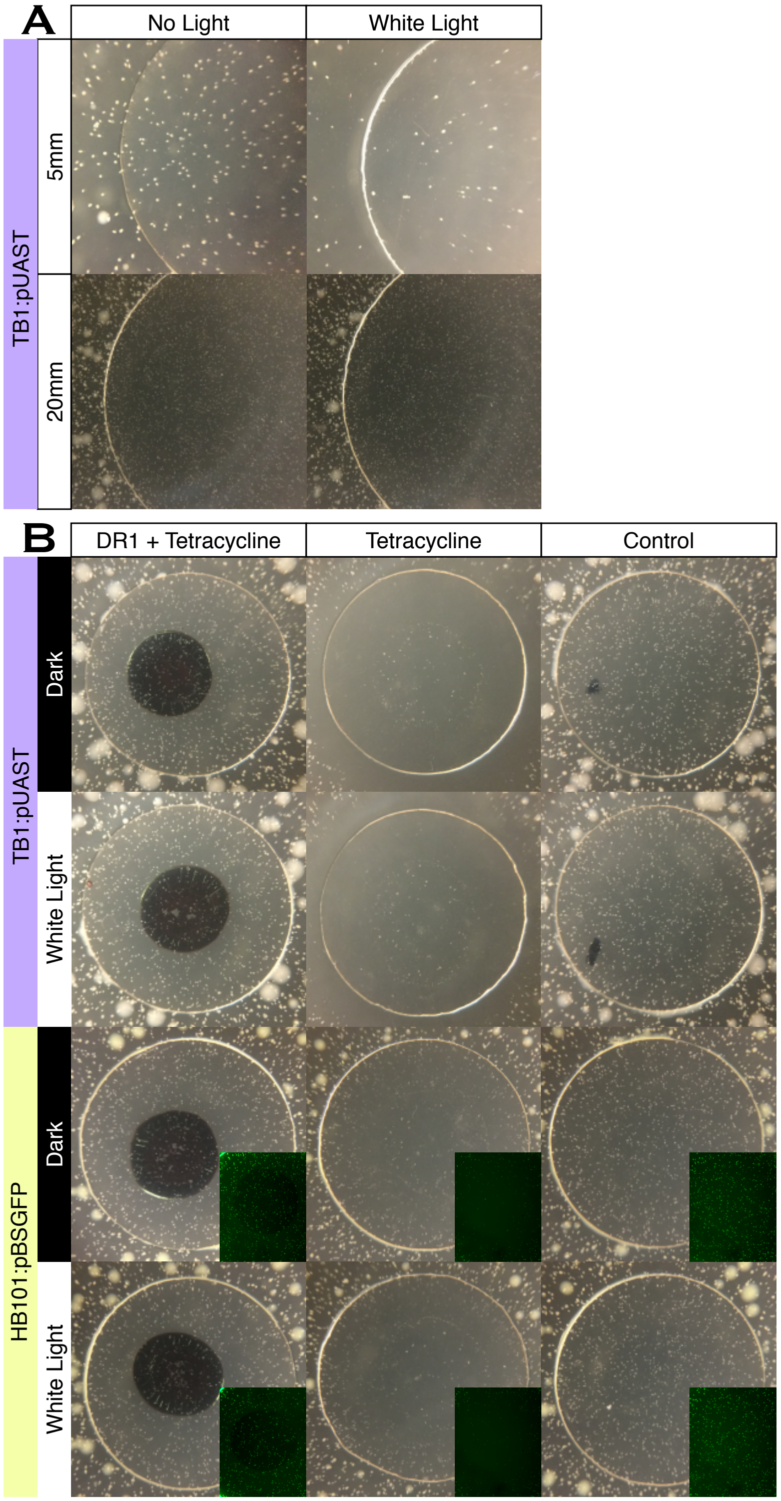

Figure S5: Tetracyline cannot be released with white light.

A. Unlike green light, white light generates heat that can inhibit bacterial growth. The agar diffusion assay was performed with blank coverslips and was incubated with or without exposure to white light. When the light is placed 5 mm (top) above the coverslip, most of the bacteria are unable to grow. In the next experiment (bottom), the bacterial density was increased and the light placed 20mm above the coverslip. At this distance, the light has no effect. **B.** A release test using coverslips with 600 μg of DR1/PMMA and 0.625 μg of tetracycline was performed with the white light placed 20mm above the coverslips. There is no observable release of tetracycline by exposure to white light.

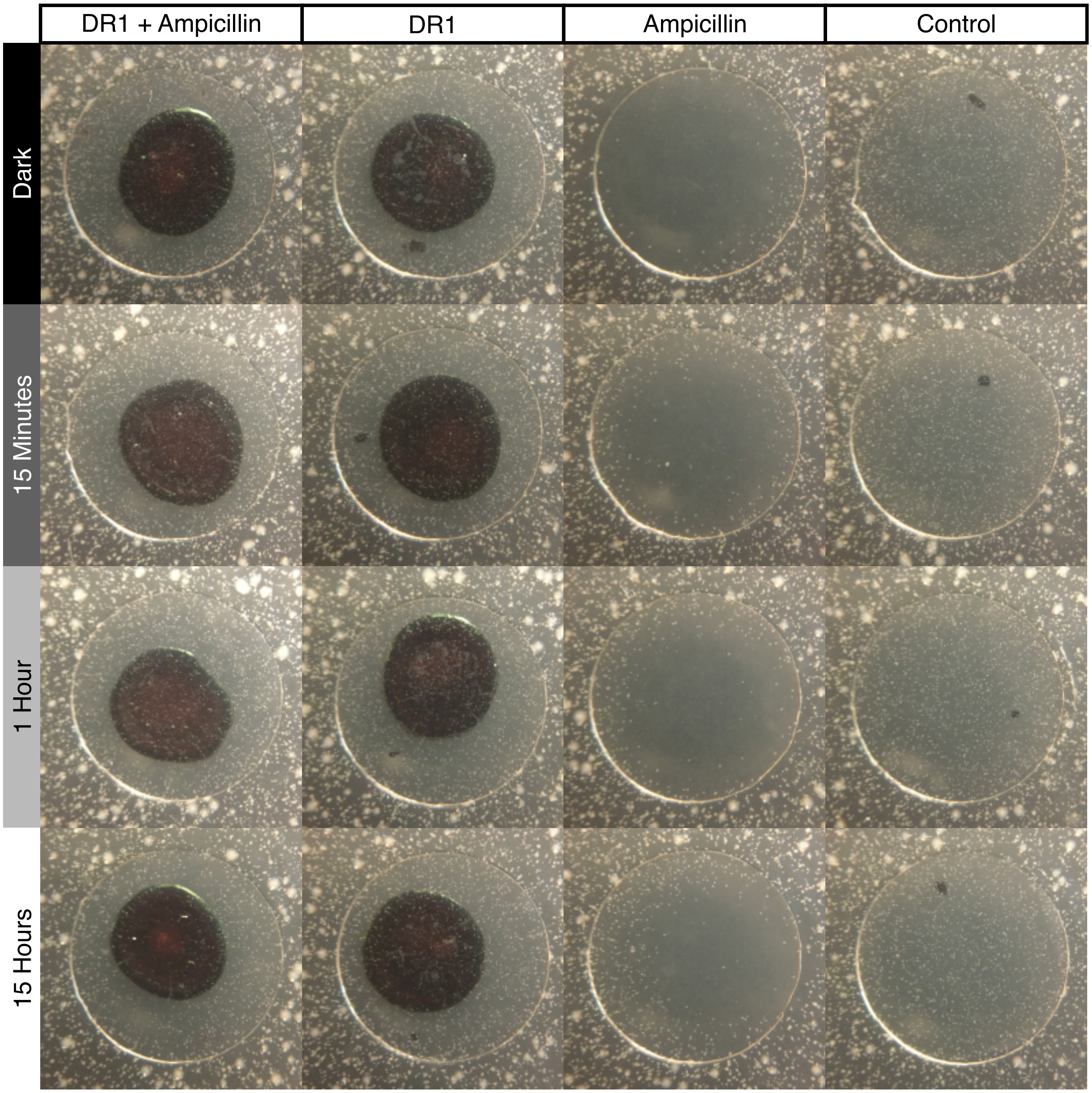

Figure S6: Timing of release of ampicillin

Exposure duration required for antibiotic release by DR1.

The agar diffusion assay was performed with the XL1Blue strain. The DR1 + Ampicillin and DR1 coverslips have 600μg DR1/PMMA. The DR1 + Ampicillin and Ampicillin coverslips have 2.5μg ampicillin. The incubation was done without light (Dark) or with the green light atop the coverslips for the indicated amount of time at the beginning of the incubation (15 minutes, 1 hour) and for the whole duration of the incubation (15 hours). The release of ampicillin is not detectable with up to one hour exposure.
